# The Amalgam Question

**Published:** 1882-07

**Authors:** J. B. Hodgkin


*The Amalgam Question.*—I am somewhat perplexed about the amalgam question, or rather the injurious effects of mercury as used in dentistry. Some weeks ago there was published in the newspapers an account of Senator Hill's troubles, the probable cause of which was attributed to amalgam fillings in his teeth.

I have been asked a good many times since if amalgam fillings were injurious, and have given a negative reply. To my sur-surprise in looking over the proceedings of the American Medical Association (in the *Virginia Medical Monthly*, of June '82, page 188, section on Dentistry,) it gives amalgam fillings the name of being and doing some very bad things. If the six conclusions as presented by Dr. Talbot be true, I am very much in the dark, and would like to have more light on this subject.

I have not seen the deleterious effects spoken of, nor do I want to see them. If I am poisoning people by putting amalgam in their teeth I want to stop it at once. I have my doubts about the mercury in amalgam fillings producing poison, also red rubber plates. The worst mouth that I have ever seen was produced by a badly fitting *gold* plate. If you can give me any information on this subject I will be much obliged.

ENQUIRER.

That this qustion of amalgam is taken up with apathy by the writer, is unquestionable. It is getting hackneyed. If Dr. Talbot saw all he professes to have seen, then no one can blame him for sounding this loud alarm. But did he? It was the German poet Gœthe we think who said that "the eye sees what it caries with it the capacity to see," and too often it is the case that the eye sees only what it *desires* to see. The truth is probably that the writer of the paper read before the "section of Dentistry" saw a chance to make a sensation before a body of men whose fears were naturally easily excited on such a subject, and taking his stand, *per saltem*, he proceeded to gather around him the "facts," seeing them with a desire to see them just so.

It is doubtful if Dr. Talbot would have the courage to read such a paper before any reputable dental association, and if so, and he be at all sensitive, he would speedily wish to be elsewhere.

The wish to find out the *wherefore* is most laudable, but we feel perfectly sure that many of the cases of so-called mercurial or arsencial poisoning, are discovered so to be simply because the searchers had made up their minds beforehand that these *were* cases of so-and-so. What has become of the torturing neuralgias caused by contact of gold with amalgam? The writer honestly thought he had seen some such cases,—thought so because he was taught that this trouble must inevitably occur under such circumstances. Now almost daily he sees gold fillings "patched" with amalgam, and proximal fillings of the latter put in against gold grinding surface fillings, and no trouble results. The preceptor of the writer was so uncompromising a foe to amalgam as to cut out any such filling, declaring it unfit for use, and that on the ground that it would not preserve teeth! He was among the most conscientious dentists we ever knew. But his eye only carried with it the power to see the amalgam failures. At the last, and a little before his death he said sadly, "I shall have to come to it," meaning amalgam, but he died first. Oh! the talk that is in this world, founded on a few illy observed and improperly applied facts! Our correspondent may take comfort. Only this day the writers' eye fell on a newspaper paragraph that Senator Hill's malady was brought on by smoking, and a few days ago he saw it stated that it was caused by a ragged edge of a tooth abrading the tongue. And yet what have these savants to say of the multitudes of cases of idiopathic cancer, of cancer of internal organs, cancer of the liver, stomach, rectum, testicle, brain concur where it is impossible for etxernal cause to be the producing factor? Meantime our correspondent can go on and make as many amalgam fillings as he cannot do well with gold, and keep a clear conscience as to mercurial poisoning, which, so far as fillings is concerned is mercurial nonsense. If he could step suddenly into Dr. Talbots' office we have little doubt that he would surprise him in *flagrante delicte,* the very act of poisoning his patient, apologising for it by saying that "*he* was very careful, only using the purest materials," etc.

J. B. Hodgkin.

